# Psychological Profiling of Triathlon and Road Cycling Athletes

**DOI:** 10.3389/fpsyg.2018.00825

**Published:** 2018-07-04

**Authors:** Aurelio Olmedilla, Gema Torres-Luque, Alexandre García-Mas, Victor J. Rubio, Eugenio Ducoing, Enrique Ortega

**Affiliations:** ^1^Department of Personality, Evaluation and Psychological Treatment, Universidad de Murcia, Murcia, Spain; ^2^Department of Didactics of Musical, Plastic and Body Expression, Universidad de Jaén, Jaén, Spain; ^3^Department of Basic Psychology, Universitat de les Illes Balears, Palma, Spain; ^4^Department Biological and Health Psychology, Universidad Autonoma de Madrid, Madrid, Spain; ^5^School of Physical Activity, Sports, and Health Sciences, University of Santiago de Chile, Santiago, Chile; ^6^Department of Physical Activity, Universidad de Murcia, Murcia, Spain

**Keywords:** stress, performance, motivation, mental skills, sport

## Abstract

Psychological characteristics of athletes play a key role in sport performance and may moderate and mediate the influence of technical, tactical, and physical abilities athletes show. Different authors have emphasized the special attention such psychological characteristics should receive considering the extent they can influence athletes’ behavior either in training or in competition. This paper is aimed at describing the psychological profiles of two cycling sports: triathlon and road cycling. One hundred and twenty-nine male and female professional and amateur cycling athletes (35.74 years old average age ±12.79; 14.94 average number of years practicing cycling ±11.20) were assessed on different psychological characteristics. For that purpose, the Psychological Characteristics related to the Sport Performance (CPRD) Questionnaire and the Psychological Skills Inventory for Sports (PSIS) was used. Results showed significant differences among triathlon and road cyclists (Stress control = *t*_116_ =-3.711, *p* = 0.000, *d* = 0.48 ; Influence of Performance Evaluation = *t*_115_ =-3-115, *p* = 0.002, *d* = 0.49; Motivation = *t*_124_ =-5.520, *p* = 0.000, *d* = 0.82; Mental Skills = *t*_119_ =-4.985, *p* = 0.000, *d* = 1.02). There were no significant differences between men and women though there were differences among pros and amateur athletes. Triathlon professional, compared to amateurs, showed higher scores in all the psychological dimensions assessed (Stress control = *t*_85_ = 3.005, *p* = 0.003, *d* = 1.07; Influence of Performance Evaluation = *t*_83_ = 2.858, *p* = 0.005, 0.77; Motivation = *t*_91_ = 2.721, *p* = 0.008, *d* = 0.26; Mental Skills = *t*_87_ = 2.556, *p* = 0.012, *d* = 0.77). The results of this descriptive study contribute to establishing a model of optimal psychological profiling applied to the different cycling groups that can be used by sport psychologist, trainers, and coaches in order to promote peak performance of these athletes.

## Introduction

Nowadays it is well known that psychological variables are quite significant aspects in sports performance, both in elite and amateur athletes (Morris, 2000; MacNamara et al., 2010; Castilla and Ramos, 2012; Abdullah et al., 2016; Swann et al., 2017). The role of psychological characteristics is relevant not only due to its direct impact on athlete’s performance (e.g., coping with or choking under stress), but also as a mediator between the athlete’s physical, technical, and tactical skills and his/her performance in competition, whether positively or negatively (Mahamud et al., 2005; Anderson et al., 2014; Arthur et al., 2017). In this vein, some seminal studies found that the physiological variables accounted for between 45 and 48% of the sport performance, but when psychological variables were added, the percentage of variance explained rose to about 79% and 85% in sports such as wrestling (Nagle et al., 1975; Silva et al., 1981; James et al., 2016).

There have been different approaches to the study of the role of psychological variables in sports performance. For instance, several authors analyzed the role of athletes’ personality traits on their sport performance (Mahoney and Avener, 1977; Gould et al., 1981; Burnik et al., 2005; Rasmus and Kocur, 2006; Cabrita et al., 2014). That is the case with Gee et al. (2007), who conducted a piece of research over 15 years with professional ice hockey players in North America (NHL) and showed that competitiveness, self-confidence, and analytical disposition were significant predictors of the athletes’ performance.

Likewise, some others focused on the differences in such variables between athletes (even those who do not compete in a regular basis) and non-athletes. For instance, Schurr et al. (1977) found team athletes were more extroverted, less dependent, with higher abstract reasoning and higher strength of self than non-athletes, and individual-sport athletes showed greater objectivity, greater dependence, less anxiety and more abstract thinking than non-athletes. More recently, Malinauskas et al. (2014) found higher Conscientiousness scores in athletes compared to non-athletes and higher Extraversion scores in team-sport athletes compared to individual-sport athletes. Similarly, Steca et al. (2018) found athletes who had experienced the most success in their careers showed higher scores in all big-five personality dimensions but Openness to Experience compared to non-athletes, and in all but Extraversion and Agreeableness compared to the less successful athletes. Moreover, they also found that individual-sport athletes were more energetic and open than team-sport athletes. In the same vein, Laborde et al. (2016) found athletes, compared to non-athletes, and individual-sport athletes, compared to team-sport athletes, scoring higher in positive personality trait characteristics. These results were in line with previous results these authors have found regarding mental toughness, athletes being tougher than non-athletes (Guillén and Laborde, 2014).

Despite all these efforts and results, evidence does not clearly support a specific personality profile distinguishing athletes from non-athletes (Weinberg and Gould, 2014). Moreover, such approaches have been also criticized by practitioners and applied researchers due to the scant usefulness of the information obtained. A more recent approach has focused on the study of mental strategies, skills, and behaviors that athletes use to compete and their relationship with the athletes’ performance (Romero et al., 2010; Álvarez et al., 2014; García-Naveira et al., 2015).This approach has been able to provide a better knowledge of the most relevant psychological characteristics to athletes’ performance as well as to observe the differences between different sports’ practitioners and between different players’ tactical positions in the same sport (Olmedilla et al., 2015, 2017).

From an applied point of view, knowing athletes’ psychological skills may allow establishing working hypotheses about the most appropriate psychological intervention to promote sports performance (Gimeno et al., 2007; Olmedilla et al., 2010, 2013; Abenza et al., 2014), not only to professional or peak-performance athletes, but also to amateurs and young trainees (Carmona et al., 2015).

Moreover, there are emerging sports that are currently congregating a large number of practitioners which had not drawn to much research attention till now (López-Cazorla et al., 2015). Additionally, some of these emerging sports such as trail-running, jogging, cycling, or triathlon do not have clear boundaries between competitive and amateur athletes, compare to the conventional sport distinction. The emergence of these sports and the lack of previous research over them, together with the request of an empirically-based recommendations to coaches and athletes for individualizing and optimizing training programs and improving sport performance, demand systematic studies to carry out (Reigal et al., 2018). Therefore, the objective of this study is to analyze the psychological characteristics (stress control, influence of performance evaluation, motivation and mental skill) related to sports performance in cyclists and triathletes.

## Materials and Methods

### Participants

One-hundred and twenty-nine cyclists and triathletes (69% male and 31% female) voluntary participated in the study. Mean age was 30.74 ± 8.79 years and mean of years practicing the sport was 14.94 ± 8.20 years. Up to 74.4% of the sample were triathletes and 25.6% were cyclists. Regarding professionalism, 27.1% were pros and 72.9% were amateurs.

### Measures

Psychological variables were assessed using the Psychological Characteristics Related to Sport Performance (CPRD, Gimeno et al., 2001) Questionnaire (see **Table 1**), based on the Psychological Skills Inventory for Sports (PSIS, Mahoney et al., 1987). The questionnaire consists of 55 items graded in a 5-option Likert scale (from totally disagree to totally agree). It also includes a response option “I do not understand” to avoid missing answers.

**Table 1:** Psychological characteristics related to the sport performance questionnaire (CPRD).

Items	I totally disagree			I totally agree
1. I usually have difficulties in concentrating when competing				
2. I usually keep thinking about the next competition or match over and over when I go to bed				
3. I really trust in my technical skills				
4. Sometimes I don’t feel like going to the training				
6. I seldom feel as tense as for interfering with my performance				
7. Frequently I mentally rehearse my performance immediately before starting my competition or match				
8. In most of competitions or matches I believe I will get it right				
9. When I fail, I usually lose my momentum				
10. It doesn’t take much to undermine the confidence in myself				
12. I’m usually scared to death immediately before starting my competition or match				
13. When I fail, it’s hard to me to refocus on what I have to pay attention to				
14. Any mild injury or bad training might erode the confidence in myself				
15. I set goals or objectives to pursue and usually I get them				
16. Sometimes I feel extremely uneasy when I am competing				
17. During my competition or match performance, my attention shifts again and again from the athletic performance and other things				
19. I usually doubt myself regarding getting it right when competing or playing a match				
20. I spend great deal of energy trying to keep calm before a match or a competition				
21. When I start off crooked, my confidence quickly decreases				
23. When I mentally rehearse my performance I see myself as if I was watching me on a TV screen				
24. I usually keep playing confidently whether I’m playing really bad				
25. When facing a match or a competition I try to picture from my own perspective what I will see, do and feel when the situation become real				
26. Confidence in myself is rather unstable				
28. I feel very nervous when I fail in a match or a competition				
29. At this moment, doing well in sport is my main focus				
30. I’m good controlling my anxiety				
31. Sport is my life				
32. I believe in myself				
33. I’m usually motivated to improve day by day				
34. I frequently lose my mental focus during the competition or the match due to referees or umpires wrong decisions against me or us				
35. When I fail during a match or a competition, I’m usually concern about what others might think (coach, mates, spectators)				
36. The day before a match or a competition I’m usually worried or anxious				
37. As a rule of thumb, I set goals which are 100% achievable regardless the others				
39 It’s not worth to spend so much time and effort I put into sport				
40. I usually psych up when dealing with matches or competitions				
41. During a match or a competition, I often lose my focus due to I worry about the final score or mark				
42. I usually take criticism easily and try to learn from them				
43. I’m easily focused on what is really important at any time during a match or a competition				
44. I can hardly accept giving prominence to some other teammate’s contribution despite mine				
45. When the match or the competition was finished, I objectively and specifically analyze my performance according to facts and the different match/competition phases				
46. I often lose my focus due to competitors’ trash talking				
47. I’m really concern about coach’s match/competition decision regarding me				
48. I don’t mentally rehearse as an ordinary routine, something I should improve				
49. I am usually very focused during trainings				
50. I frequently set main goals before each training session or competition/match				
51. My confidence regards on previous competition or matches success				
52. My motivation regards on receiving recognition from others				
53. Coach’s instructions, comments and gestures often negatively affects my concentration during matches or competitions				
54. I usually feel confident in myself even in tough matches or competition situations				
55. I’m willing to any effort to improve myself				

^1^*Buceta, J. M., Gimeno, F., and Pérez-Llantada, M. C. (1994). Based on the Psychological Skills Inventory for Sport (PSIS), Mahoney et al. (1987)*.

CPRD includes five subscales: Stress Control (SC), Influence of Performance Evaluation (IPE), Motivation (M), Team Cohesion (TCOH), and Mental Skills (MSK), showing acceptable values of internal consistency for the total scale (α = 0.85) and for most of the subscales (α_SC_ = 0,88; α_IPE_ = 0,72; α_M_ = 0,67; α_TCOH_ = 0,78; α_MSK_ = 0,34). According to the authors, the low internal consistency of MSK is probably related to this dimension tapping a wide range of different skills but they kept the subscale due to the factorials loads showed by the items of this factor (over 0.30, Gimeno and Pérez-Llanta, 2010). Moreover, other studies that used the original PSIS had got similar results regarding that subscale (Chartrand et al., 1992). TCOH subscale has not been used in this study due to the nature of the targeted sports.

### Procedure

After the corresponding author’s institution IRB approval (UM1551/2017), athletes were connected by cycling and triathlon sport clubs. The researchers informed athletes about the objectives and use of the information and those who voluntarily participated signed an informed consent.

### Data Analysis

*T*-test for independent samples were used to test differences between sport modality (cycling vs. triathlon), gender and level (pro vs. amateur) regarding to the different psychological variables.

In addition to the univariate analyses, the psychological measures of the athletes between sports (cycling and triathlon), gender (men and women), and categories (i.e., professional and amateur) were compared by the method of magnitude-based inferences (MBI) (standardized Cohen’s *d* values and their 90% Confidence Intervals). The comparison on each psychological measure was done using the Hopkins’ spreadsheet with the smallest worthwhile difference (Batterham and Cox, 2006). This method calculated 0.2 times the standardization, estimated from the between-subjects standard deviation. According to the authors (Hopkins et al., 2009) the differences can be defined as unclear if the confidence intervals for the difference in the means included substantial positive and negative values (± 0.2^∗^standardization) simultaneously. In order to assess the differences between pairs of comparisons, the magnitude of a clear difference was assessed as follows: >0.25, trivial; 0.25–75% possibly, 75–95% likely, 95–99% very likely, >99% most likely (Hopkins, 2007). All data analyses were carried out using the SPSS v. 21.0 Statistical package.

## Results

**Table 2** shows means and *SD*s of each one of the psychological variables assessed according to sport modality as well as inferential statistics.

**Table 2:** Mean, *SD*, *p* value and effect size of each psychological variable according to sport modalities.

	Clycling (*n* = 33)	Triathlon (*n* = 96)	Total (*N* = 129)	*P* value	ES	MBI
SC	28.68 ± 16.70	42.68 ± 18.47	39.00 ± 19.00	0.000	0.48 (0.16–0.80)	Possibly
IPE	18.81 ± 9.39	25.94 ± 11.58	23.99 ± 11.44	0.002	0.49 (0.16–0.32)	Possibly
M	11.24 ± 5.55	17.81 ± 5.97	16.09 ± 6.52	0.000	0.82 (0.51–1.12)	Likely
MSK	12.41 ± 5.49	19.99 ± 7.93	17.98 ± 8.08	0.000	1.02 (0.68–1.37)	Very Likely

*SC: Stress control; IPE: Influence of Performance Evaluation; M: Motivation; MSK: Mental Skills; ES = Effect size values for standardised Cohen’s d; MBI = magnitude based inference effects*.

As can be seen, triathletes scored significantly higher than cyclists in the four psychological dimensions studied (SC = *t*_116_ = -3.71, *p* = 0.000; IPE = *t*_115_ = -3.11, *p* = 0.002; M = *t*_124_ = -5.52, *p* = 0.000; MSK = *t*_119_ = -4.98, *p* = 0.000). In addition, qualitative probabilistic inference according to MBI resulted in the differences in Mental Skills being as very likely (95–99% probability of the effect being substantially positive), the differences in Motivation being likely (75–95%), and the differences in both Stress Control and Influence of Performance Evaluation being possible (25–75%)

**Tables 3**, **4** shows the results regarding gender and sport modality.

**Table 3:** Mean, SD, *p* value and effect size of each psychological variable according to sport modalities and gender in Cycling.

Subscale	Cycling				
	Males	Females	ES	MBI	*P*-value
Stress Control	26.00 ± 16.62	37.86 ± 14.47	0.51 (0.07–0.96)	Possibly	0.099
Influence of Performance Evaluation	17.63 ± 10.10	22.38 ± 6.05	0.42 (0.04–0.80)	Possibly	0.221
Motivation	11.16 ± 5.40	11.50 ± 6.39	0.04 (-0.65–0.72)	Possibly	0.883
Mental Skills	11.92 ± 4.56	14.14 ± 8.28	0.27 (-0.68–1.21)	Possibly	0.353

*ES: Effect size values for standardised Cohen’s d; MBI = magnitude based inference effects*

**Table 4:** Mean, SD, *p* value and effect size of each psychological variable according to sport modalities and gender in Triathlon.

Subscale	Triathlon				
	Males	Females	ES	MBI	*P*-value
Stress Control	44.16 ± 19.25	40.13 ± 17.07	0.24 (-0.11–0.58)	Possibly	0.328
Influence of Performance Evaluation	26.41 ± 11.70	25.03 ± 11.50	0.27 (-0.10–0.64)	Possibly	0.606
Motivation	17.52 ± 5.99	18.39 ± 6.00	-0.19 (0.23–0.42)	Possibly	0.511
Mental Skills	20.30 ± 7.71	19.44 ± 8.42	0.10 (-0.28–0.48)	Possibly	0.626

*ES: Effect size values for standardised Cohen’s d; MBI = magnitude based inference effects*.

Results show there were no statistically significant differences between men and women either in cycling (SC = *t*_29_ = -1.70, *p* = 0.099; IPE = *t*_30_ = -1.25, *p* = 0.221; *M* = *t*_31_ = -0.15, *p* = 0.883; MSK = *t*_30_ = -0.94, *p* = 0.353) or in triathlon (SC = *t*_85_ = 0.98, *p* = 0.328; IPE = *t*_83_ = 0.52, *p* = 0.606; *M* = *t*_91_ = -0.66, *p* = 0.511; MSK = *t*_87_ = 0.49, *p* = 0.626) in any of the psychological characteristics. Additionally, the results of the MBI showed the probability of the effect being substantially positive just possible for all the variables.

**Tables 5**, **6** shows the results according to professional level (pros vs. amateurs) in both modalities.

**Table 5:** Mean, SD, *p* value and effect size of each psychological variable according to sport modalities and professional level in Cycling.

Subscale	Clycling				
	Professional	Amateur	ES	MBI	*P*-value
Stress Control	33.57 ± 17.49	27.25 ± 16.58	-0.01 (-0.67/0.65)	Possibly	0.387
Influence of Performance Evaluation	21.75 ± 4.10	17.83 ± 10.48	-1.83 (-3.19/–0.47)	Very Likely	0.315
Motivation	8.89 ± 5.18	12.13 ± 5.53	0.45 (-0.13/1.03)	Possibly	0.315
Mental Skills	11.44 ± 3.00	12.78 ± 6.23	0.09 (-0.65/0.84)	Possibly	0.545

*ES: Effect size values for standardised Cohen’s d; MBI = magnitude based inference effects*.

**Table 6:** Mean, SD, *p* value and effect size of each psychological variable according to sport modalities and professional level in Triathlon.

Subscale	Triathlon				
	Professional	Amateur	ES	MBI	*P*-Value
Stress Control	52.17 ± 14.56	39.27 ± 18.64	1.07 (-0.54/1.59)	Very Likely	0.003
Influence of Performance Evaluation	31.77 ± 23.90	23.90 ± 11.76	0.76 (0.28/1.25)	Likely	0.005
Motivation	20.42 ± 16.79	16.79 ± 5.36	0.26 (0.13/0.64)	Possibly	0.008
Mental Skills	23.52 ± 18.76	18.76 ± 8.14	0.77 (0.31/1.23)	Likely	0.012

*ES: Effect size values for standardised Cohen’s d; MBI = magnitude based inference effects*.

*T*-tests showed no significant differences between professional and amateur cyclists in any of the variables analysed (SC = *t*_29_ = 0.88, *p* = 0.387; IPE = *t*_30_ = 1.022, *p* = 0.315; *M* = *t*_31_ = -1.52, *p* = 0.315; MSK = *t*_30_ = -0.61, *p* = 0.545). However, MBI results showed professional cyclists scoring very likely higher in Influence of Performance Evaluation than amateurs and possibly higher in Stress Control but possibly lower in Motivation and Mental Skills (see **Table 5**).

Conversely, professional triathletes scored higher than amateurs in all the variables explored (see **Table 6**) (SC = *t*_85_ = 3.00, *p* = 0.003; IPE = *t*_83_ = 2.86, *p* = 0.005; *M* = *t*_91_ = 2.72, *p* = 0.008; MSK = *t*_87_ = 2.56, *p* = 0.012). Moreover, the results of the MBI reflected the relevance of these differences. There were a substantial difference in Stress Control (95–99% probability), a large difference in Influence of Performance Evaluation and Mental Skills (75–95% probability), and moderate difference in Motivation (25–75%).

## Discussion

This study has explored the psychological profile of two different endurance sports, cycling, and triathlon, and analyzed whether gender and professionalism level present differences in athlete’s core psychological characteristics. The results of this study have shown there are differences in the psychological characteristics explored between modalities and between level of professionalism. Unlike other studies that have not clearly found differences between practitioners of these two sports (see Schurr et al., 1977), this study did find significant differences in all the variables studied: triathletes scored higher than cyclists in all the variables with an important size of effect in two of them: the athletes’ mental skills and their motivation.

Whether the differences between the two sport modalities are due to the difference in the nature of the two sports more than the differences between the practitioners is still open. Triathlon embeds cycling, and in triathlon competitions, perhaps for some athletes the cycling competition is instrumental, and only make sense in conjunction with the other two swimming and running parts. Eventually, these results are in line with Reigal et al.’s (2018) who compared triathlon to some other sports modalities in mental skills and found differences not just only between triathletes and soccer and golf players but also between triathletes and track and field athletes.

An explanation might be related to the fact that while the training and preparation schedules may be similar, the situations and competitions are very different, cycling being much more tactical than triathlon. If the physical differences, not the tactics, tip the balance towards the side of the triathletes, perhaps the same should happen with the psychological ones, among which the mental ability and the motivation to persist in the sport stand out. Moreover, the reduced sample size of cyclists demands to be cautious.

Regarding gender, this study has shown that in these two sports, there are no differences among men and women regarding psychological variables. This result is in agreement with recent work in the same line (Hanton et al., 2009). Nevertheless, we must be cautious before extrapolating the results. For example, although no differences have been found regarding the competitive anxiety when is considered globally, in other studies we have found clear differences between the two genders when analyzing the factor of competitive anxiety related to the worry for the athletes’ performance (Ponseti et al., 2017).

In line with a possible blurring between the professional and amateur categories in these two sports, no differences have been found in cycling. Although professional Stress Control and Influence of Performance Evaluation scores were higher – this one showing with a relevant MBI score – there were no statistically significant differences supporting a possible subdivision of the variables studied by the CPRD in these two blocks.

Conversely, professional triathletes clearly showed significant differences in all psychological variables, SC and IPE being particularly likely according MBI values. These results are perfectly compatible with the competitive restrains. Again, the same scheme is repeated, and perhaps should be with the same explanations, as when the data are considered globally regarding the differentiation between the two sports.

The results of this study provide relevant information regarding the psychological characteristics of the practitioners of these two sports, cycling and triathlon, and such psychological profiling may be a useful tool for designing general psychological training, and/or specific interventions (Olmedilla et al., 2013).

### Limitations and Future Research Directions

As with any cross-sectional study, the lack of repeated measures to determine stability across the different competitive and training situations should be taken into account and future studies should include follow-up assessment which might be able to capture the effects of situational and transitional events the athlete has to cope with.

In addition, we must bear in mind that this descriptive study did not include an objective assessment of sport performance, which should also be considered in the future. Finally, there should be noted that there is a reduced cyclist sample size. Therefore, generalizations should be made cautiously.

## Conclusion

(1) There are significant differences between cyclists and triathletes, with respect to all the variables studied with the CPRD.
(2) No gender differences have been found with respect to sports practiced, nor in terms of psychological variables.
(3) There are some differences between amateur and professional practitioners in psychological characteristics, but they did not show a consistent pattern among the two modalities, except for stress management and performance evaluation.

## Ethics Statement

This study was carried out in accordance with the recommendations of the Declaration of Helsinki. The protocol was approved by the Comité de Ética de la Universidad de Murcia (reference: UM 1551/2017). All subjects gave written informed consent in accordance with the Declaration of Helsinki.

## Author Contributions

AO, AG-M, and EO contributed with the conception and design of the study. ED organized the database. ED and EO performed the statistical analysis. AO wrote the first draft of the manuscript. VR, AG-M, GT-L, and EO wrote sections of the manuscript. All the authors contributed to the revision of the manuscript, and read and approved the presented version.

## Conflict of Interest Statement

The authors declare that the research was conducted in the absence of any commercial or financial relationships that could be construed as a potential conflict of interest.
